# Protocol for an economic evaluation of a tele-neurologic intervention alongside a stepped wedge randomised controlled trial (NeTKoH)

**DOI:** 10.1186/s12913-023-09985-5

**Published:** 2023-09-22

**Authors:** Ana S. Oliveira Gonçalves, Imke Mayer, Ricarda S. Schulz, Agnes Flöel, Felix von Podewils, Anselm Angermaier, Kerstin Wainwright, Tobias Kurth, the NeTKoH Consortium, Paula J. Filser, Aiham Alkhayer, Verena Horn, Wieland Köhn, Malgorzata Kotarz-Boettcher, Anne Krüger, Cordula Weil, Carl Witt, Jean-Francois Chenot, Simone Kiel, Elisa Michalowsky, Michael Böttcher, Diana Graja, Katrin C. Reber, Olga Resch, Juliane Rothe, Jacqueline Syring

**Affiliations:** 1 Institute of Public Health, Charité – Universitätsmedizin Berlin, Berlin, Germany; 2 Department of Neurology, University Medicine Greifswald, Greifswald, Germany

**Keywords:** Tele-neurology, Telemedicine, Primary care, General practitioner, Health care management, Cost-effectiveness, Cost-utility, Statutory health insurance, Claims data, Cluster randomized trial

## Abstract

**Background:**

A significant and growing portion of the global burden of diseases is caused by neurological disorders. Tele-neurology has the potential to improve access to health care services and the quality of care, particularly in rural and underserved areas. The economic evaluation of the stepped wedge randomised controlled trial NeTKoH aims to ascertain the cost-effectiveness and cost-utility regarding the effects of a tele-neurologic intervention in primary care in a rural area in Germany.

**Methods:**

This protocol outlines the methods used when conducting the trial-based economic evaluation of NeTKoH. The outcomes used in our economic analysis are all prespecified endpoints of the NeTKoH trial. Outcomes considered for the cost-utility and cost-effectiveness analyses will be quality-adjusted life years (QALYs) derived from the EQ-5D-5L, proportion of neurologic problems being solved at the GP’s office (primary outcome), hospital length-of-stay and number of hospital stays. Costs will be prospectively collected during the trial by the participating statutory health insurances, and will be analysed from a statutory health insurance perspective within the German health care system. This economic evaluation will be reported complying with the Consolidated Health Economic Evaluation Reporting Standards (CHEERS) checklist.

**Discussion:**

This within-trial economic evaluation relaying the costs and outcomes of an interdisciplinary tele-consulting intervention will provide high-quality evidence for cost-effectiveness and policy implications of a tele-neurological programme, including the potential for application in other rural areas in Germany or other jurisdictions with a comparable health system.

**Trial registration:**

German Clinical Trials Register (DRKS00024492), date registered: September 28, 2021.

**Supplementary Information:**

The online version contains supplementary material available at 10.1186/s12913-023-09985-5.

## Background

Although age-standardised incidence, mortality, and prevalence rates of many neurological disorders have been declining, the burden of neurological disorders, as measured by the absolute number of disability-adjusted life years (DALYs), keeps increasing [1]. In 2017, neurological disorders were the third main contributor of DALYs in the European Union (13.3%), right after cardiovascular diseases, and cancer. This can be explained by the long life expectancy in these countries, as well as by the increasing incidence, and the increasingly long duration of ageing-related diseases [2]. In 2010, the costs associated with brain disorders (mental and neurological disorders) in Europe amounted to €798 billion. The high costs of brain disorders were associated with €295 billion in direct health costs, €186 billion in non-medical costs (nursing homes, etc.), and €315 billion in indirect costs (absenteeism from work, pensions, etc.) [3].

Nonetheless, patients suffering from neurological disorders often face multiple hurdles to visit a specialist. While patients in Germany can easily visit a general practitioner (GP), visiting specialists is normally associated with considerable waiting times and logistical difficulties [4, 5]. This problem is more pronounced in rural areas. In the past, the number of neurologists per 100,000 inhabitants in the state of Mecklenburg-West Pomerania was below the national average [6]. Furthermore, there is a wave of outpatient specialists soon reaching retirement age, thus jeopardising the care of the local population [7].

Telemedicine has the potential to deliver health care services to both remote and underserved communities [7, 8]. It can be defined as “the use of telecommunications technology for medical diagnosis and patient care” [9]. Moreover, it has revolutionised inter- and multidisciplinary meetings, where physicians in different medical fields and supporting medical staff can interact. These meetings through telemedicine can foster access to expert opinion, and reduce time for diagnostics and treatment [10]. Telemedicine used in the context of neurological disorders (henceforth “tele-neurology”) can prove to be a powerful tool to strengthen the availability, affordability, and convenience of care for patients in these communities [11].

Multiple studies have analysed the (clinical) effectiveness of delivering telemedicine [12]. However, it is also important to take into account whether telemedicine services represent a good use of funds, and whether healthcare systems should reimburse them. Limitations on billing and reimbursement for time spent in teleconsultations, large investment costs in equipment, bandwidth requirements, and lack of trained employees to use the equipment are mentioned as barriers to the implementation of telemedicine [8, 13]. Thus, it is important to weigh the outcomes of telemedicine interventions and their associated costs against each other by conducting economic evaluations. A recent umbrella review of clinical and cost-effectiveness telemedicine trials assessed eighteen reviews and found evidence that telemedicine can be cost-effective [14]. However, none of the included systematic reviews focused on neurological disorders, and the methodological quality of the majority of the systematic reviews included was deemed as low or critically low, thus hindering the generalisability of their findings.

The NeTKoH trial will assess the use of tele-neurology in a primary care setting in an underserved area in Germany. To investigate the economic impact of NeTKoH on the German health care system, cost-effectiveness analyses, and a cost-utility analysis will be conducted alongside the NeTKoH trial to ascertain the relationship between health outcomes and costs of this new intervention in comparison to standard care. Findings from this economic evaluation can be used as a basis for health policy-makers to facilitate decisions.

## Methods and design

### Design and study population

We will conduct the economic evaluation alongside the NeTKoH study, a cluster-randomised controlled trial with a stepped wedge design carried out in a rural area in Germany, from October 2021 to October 2024.

Briefly, NeTKoH offers patients who present themselves to their outpatient GPs with neurological symptoms and diseases in a rural area of northeast Germany to receive neurologic speciality care via a face-to-face video conferencing system between the local outpatient GP and a neurologist from a neurology department of a university clinic in the region. The patients in the control group receive standard care, which only involves in-person face-to-face visits with the local GP.

The main objective of NeTKoH is to strengthen the provision of neurologic care in outpatient GP offices in a rural area by increasing the proportion of neurologic problems being solved at the GP’s office (primary outcome). Secondary outcomes are the number of hospital stays, length-of-stay at the hospital, time until neurologic specialist appointments and diagnostics, patients’ health status and quality of life, and out- and inpatient referrals. Patients are eligible to participate in NeTKoH if they (1) are at least 18 years old, (2) present themselves with neurological symptoms (any symptoms that can warrant a referral to a specialist in neurology) to the participating GP, (3) members of any German statutory health insurance, and (4) able to give informed consent. Complete information on the study design and primary study population are detailed elsewhere [15].

### Sample size

The sample size calculation for NeTKoH was based on the primary endpoint, the proportion of neurologic problems being solved in the GPs’ offices, measured by the number of outpatient referrals (neurologist/other specialist), and inpatient referrals (hospital/other). The target sample of 1,089 will provide 78% power at a 5% significance level (two-sided) to detect a 50% improvement in the intervention group, assuming a baseline proportion of 0.2 of neurologic problems solved in the GP’s offices in the control group, and a variation coefficient of 0.30. Assuming a coefficient of variation of 0.20 a power of 81% can be achieved. In the context of cluster randomised trials in the real world context, a coefficient of variation of ≤ 0.25 is considered appropriate [16]. Full details on the sample size calculation for the primary endpoint are reported elsewhere [link to main study protocol].

Patients’ claims data can only be obtained if they belong to the two statutory health insurances that are participating in this study (AOK Nordost and Techniker Krankenkasse). In Germany, claims data from the statutory health insurances are subject to a waiting period of up to 9 months (with waiting periods for inpatient claims data being shorter than those are for outpatient claims data). Hence, it will not be possible to analyse claims data from all recruited patients belonging to these statutory health insurances. According to the current information we obtained from the consortium, we expect to recruit and be able to analyse data from 841 patients belonging to these health insurances.

Besides the participants for whom no claims data will be available and for whom the primary endpoint is missing, this economic evaluation will include all randomised patients, according to the intention-to-treat principle.

### Effects/Outcomes

For the cost-utility analysis, the outcome will be measured with the EQ-5D-5L, using German utility weights [17]. This questionnaire comprises five dimensions: mobility, self-care, usual activities, pain/discomfort and anxiety/depression, and is collected during baseline and at the 3-month follow-up. Quality-adjusted life years (QALYs) will be calculated by taking the 'area under the curve' between the baseline and 3-month follow-up assessment scores. We will control for baseline EQ-5D-5L scores [18].

The outcome measures for the cost-effectiveness analyses will include the proportion of neurologic problems being solved in the GPs’ offices (primary endpoint), the number of hospital stays, and the length of stay at the hospital. The number of hospital stays and the hospital length-of-stay can be seen as a proxy for a health condition (in general, if an individual needs to stay in the hospital, their health condition is worse than for those who do not need it). Furthermore, hospital stays and days spent in the hospital can be interpreted as a general health outcome impacting a patient's quality of life negatively (patients may feel restricted and uncomfortable during their hospital stay). These outcomes will be assessed using patient-specific claims data during the first three months after study inclusion.

### Costs

The economic evaluation will be undertaken from a German statutory health insurance perspective.

We will use claims data from two statutory health insurances (AOK Nordost and Techniker Krankenkasse) to obtain patient-specific costs. Direct medical, and direct non-medical costs, as well as indirect costs, will be considered. Direct medical costs include hospital stays, outpatient visits, drugs, therapeutic appliances, and assistive devices. Direct non-medical costs include reimbursed travel costs to medical examinations and for the indirect costs, sickness leave benefits are considered. In Germany, sick leave payments are only covered by statutory health insurances starting on the 43rd day of sickness. Up until day 42, these costs are covered by employers and are thus not considered in our analysis. We will also include costs associated with the intervention (‘intervention costs’). These include costs for training, investment costs for setting up the infrastructure, and operating costs related to the equipment. Costs associated with tele-consulting systems will be annualised over their lifetime, as provided by the firm making them available for the study. Furthermore, we will take into account staff costs related to conducting and preparing a teleconsultation, as well as the time spent with training on how to use the tele-neurology video-conferencing system. Table 1 lists the cost categories that will be considered.

**Table 1:** Cost reporting categories

Cost category	Intervention group	Control group	Source
Claims’ data from the SHI			
Inpatient care	x	x	SHI
Outpatient care	x	x	SHI
Drugs	x	x	SHI
Therapeutic appliances	x	x	SHI
Assistive devices	x	x	SHI
Transportation costs	x	x	SHI
Sickness leave benefits	x	x	SHI
Investment costs			
Inpatient and outpatient devices for tele-neurology consultations	x		Company information
Maintenance costs			
Inpatient and outpatient devices for tele-neurology consultations	x		Company information
Running costs			
Staff costs for time spent on trainings for using the tele-neurology devices	x		Collective agreements, surveys
Staff costs for time spent preparing and conducting tele-neurology consultations	x		Collective agreements, surveys

SHI—Statutory Health Insurance

All costs will be reported in euros, using 2024 as the reference year.

This economic evaluation will compare the costs and effects of each arm over the first 3 months after randomisation. Neither outcomes nor costs will be discounted, since we are only considering outcomes and costs related to the same year when they occur. The relationships between the outcomes and the costs of NeTKoH will be analysed in terms of the incremental cost-effectiveness ratios (ICER). ICERs are calculated as the difference in the mean costs between the intervention and control group divided by the difference in the mean outcomes between the intervention and control group.

### Statistical analysis

The parametric G-formula will be used to estimate incremental costs and incremental effects [19, 20].

For the costs, a generalised linear regression with a gamma distribution and log-link function will be carried out to account for the skewness of the costing data. Moreover, it will include random effects for the clusters, with the groups to be compared (intervention or control), and a priori selected covariates (age, sex, and step) as independent variables. Using this model, the expected value of the costs for each individual will be predicted for two hypothetical scenarios, in which the patient receives or does not receive the intervention. The predicted costs under each hypothetical scenario will be calculated for all patients to obtain the mean cost in each scenario (all patients in the study would have received the intervention or all patients in the study would have received the control). The difference between the mean costs in these two scenarios is then calculated to determine the incremental cost of the intervention.

A similar procedure will be used for the remaining outcomes. To calculate the incremental QALYs and incremental hospital length-of-stay caused by the intervention, both continuous variables, a linear regression will be fitted that will include the same random and fixed effects. In the regression in which QALYs is the dependent variable, we will also control for the EQ-5D-5L baseline value, as recommended in the literature [18]. For the primary endpoint (proportion of neurologic problems being solved at the GP’s office), we will run a logistic mixed regression model to predict each individual’s probability of having their case solved at the GP’s office per scenario. We will consider the same random and fixed effects as for other endpoints. Then, the probability of a case being solved will be summed across all patients for each hypothetical scenario to obtain the average number of cases resolved per scenario. Finally, the incremental average number of cases solved by the intervention will be calculated as the difference between the average number of cases solved in the intervention group and the average number of cases solved in the control group. For the endpoint number of inpatient stays, a generalised linear mixed regression with a Poisson distribution will be carried out, with the group and a priori selected covariates as independent variables. Furthermore, random effects for the clusters will be considered.

Incremental mean costs and incremental mean effects (QALYs in the cost-utility analysis and proportion of neurologic problems being solved at the GP’s office, number of inpatient stays, and hospital length-of-stay in the cost-effectiveness analysis) will be used to estimate ICERs.

Assuming a missing at random mechanism and taking into account the clustered data structure, we will conduct multilevel multiple imputation by chained equations with five datasets to impute missing values. Point estimates for incremental effects and incremental costs will be obtained as the averages across the imputed datasets. The imputation models will include as covariates the same variables included in the regression models of the primary effectiveness study, following the guidelines for handling missing data in economic evaluations [21].

### Handling uncertainty

Multiple methods will be used to deal with uncertainty: Bootstrapping, sensitivity analyses and extreme value analyses (worst-case scenario).

As a sensitivity analysis, we will conduct a logarithmic transformation of the cost variable to decrease the skewness of the data, and approximate it with a normal distribution to fit a linear mixed regression model.

Bootstrapping allows the analysis of the joint uncertainty due to sampling variation without making parametric assumptions about the distribution. We will conduct multilevel nonparametric bootstrapping according to the Boot MI method to obtain 95% confidence intervals [22]. After multiple imputation on each bootstrapped dataset, we will consider the 2.5% and 97.5% percentiles of the resulting distribution of average metric (across the 5 imputed datasets) as the confidence interval. We will also illustrate the joint uncertainty surrounding both costs and outcomes by plotting cost-effectiveness and cost-utility planes with the bootstrapped cost-effectiveness and cost-utility pairs resulting from the bootstrapping replication runs [23].

The health economic analyses will take the perspective of the German statutory health insurance. However, it is currently uncertain whether the statutory health insurance scheme would provide coverage for the tele-neurology devices used in the outpatient sector. Therefore, the effects of including this cost category will be evaluated in scenario analyses (worst-case scenario).

The reporting of this economic evaluation will follow the Consolidated Health Economic Evaluation Reporting Standards guidelines [24].

The statistical analyses will be carried out using R v4.2.1 (or higher) [25].

## Discussion

Patients residing in rural areas of Germany often encounter two major challenges when seeking care from neurologists: lengthy waiting periods and considerable travel distances. Given the current demographic trends, it is imperative to identify measures that can mitigate these issues. Tele-neurology has emerged as a viable solution, and the NeTKoH trial will conduct an economic evaluation to determine the relationship between the outcomes of a tele-neurological intervention and its associated costs. This evaluation aims to quantify the benefits of tele-neurology and provide insights into its cost-effectiveness.

Typically, an intervention is deemed cost-effective if the ICER falls below a certain threshold established by decision-makers in a specific setting. In Germany, there is currently no official threshold in place. The WHO threshold recommendations are based on the country’s gross domestic product (GDP) per capita [26]. According to these recommendations, an intervention can be considered highly cost-effective if the ICER is lower than the GDP per capita. If the ICER falls between one and three times the GDP per capita, the intervention is deemed cost-effective. However, if the ICER is higher than three times the GDP per capita, the intervention is generally considered not cost-effective. The Netherlands has a similar healthcare system (characterised by a mixed compulsory social insurance and private voluntary insurance) with an official threshold range of up to €80,000/QALY [27]. Independently of the threshold considered, we aim to present the cost-utility and cost-effectiveness results in a way that allows decision-makers to weigh up the costs and benefits and decide which interventions to adopt. We will also provide evidence-based results that can be used in the telemedicine field and, more specifically, tele-neurology.

A strength of this study is that patients’ direct medical costs, direct non-medical costs, as well as indirect costs will be obtained directly from the statutory health insurances, thus we are not dependent on patients’ self-reported data that may suffer from memory bias. Furthermore, we will use both a logarithmic transformation of the cost variable to fit a linear mixed regression model, and a generalised linear mixed model with a gamma distribution. These analyses will allow taking into account the methodological uncertainty in the modelling of costs, employing commonly used methods [28]. Moreover, our economic evaluation will include analyses with different endpoints, thus providing a more nuanced understanding of the impact of the intervention. A further strength of our study is the use of the EQ-5D-5L questionnaire to calculate utility scores. Given its widespread use, its use will allow the comparison of the NeTKoH results with other analogous (international) studies.

This planned economic evaluation has some limitations. The first involves the follow-up of the study. While we have planned the follow-up that adequately captures all relevant costs and benefits of the intervention, the follow-up may run too short. In such cases, extrapolation or modelling may be necessary to estimate long-term outcomes. However, such an approach is not feasible in this study due to the significant heterogeneity of the population under investigation. Specifically, the inclusion criteria encompass patients with diverse non-life-threatening neurological symptoms, and the small sample size precludes modelling across subgroups. Second, we will not be able to analyse claims data from all recruited patients because in Germany claims data from statutory health insurances are subject to a waiting period of up to 9 months. Hence, it will not be possible to analyse claims data from all recruited patients. Furthermore, we can only analyse claims data from recruited patients if they belong to two specific statutory health insurances (AOK Nordost and Techniker Krankenkasse). Although in total there are 97 statutory health insurances in Germany, the two insurances participating in this trial belong to different categories of statutory health insurances (Allgemeine Ortskrankenkasse—Local General Sickness Fund and Verband der Ersatzkassen—Association of Substitute Funds) [29]. Thus, we believe that the patients for which claims data will be available will be representative of the entire NeTKoH study population.

Lastly, while at baseline patients fill in their questionnaires at the GP offices, during the 3-month follow-up, questionnaires are administered by phone. Consequently, this may affect the administration of the EQ-5D-5L.

## Conclusion

NeTKoH is an interdisciplinary tele-neurological intervention that seeks to provide location-independent and cost-effective treatment to patients suffering from neurological symptoms in underserved areas of Germany. By leveraging modern telecommunication technologies, NeTKoH aims to enhance access to high-quality neurological care, which is currently limited by factors such as long travel distances and waiting times. The economic evaluation results conducted alongside this trial will yield high-quality evidence regarding the use of tele-neurology in primary care settings. The insights gained from this evaluation will be invaluable in informing the development of telemedicine strategies that can optimise the delivery of neurological care to patients in need, regardless of their geographical location.

### Supplementary Information

**Additional file 1. **

## Data Availability

Not applicable.

## Abbreviations

G-BA: Federal Joint Committee
GP: General Practitioner
NeTKoH: Neurologic Tele-Consultations in GP Practices
SHI: Statutory Health Insurance
QALY: Quality-Adjusted Life Year

## Declarations

### Trial registration and status

This study was registered at the German Clinical Trials Register (DRKS00024492) on the 28th of September, 2021. Patient recruitment started in October 2021 and will end in October 2024. Results are expected in July 2025.
