# Supplementary material for: Protocol for an economic evaluation of a tele-neurologic intervention alongside a stepped wedge randomised controlled trial (NeTKoH)
Source: BMC Health Serv Res. 2023 Sep 22;23:1021. doi: 10.1186/s12913-023-09985-5 (PMC10515046; doi:10.1186/s12913-023-09985-5)
Supplement: Supplementary file 1 — Additional file 1. [file 12913_2023_9985_MOESM1_ESM.docx]

**Supplementary File**

**The NeTKoH Consortium:**

Paula J. Filser^1^, Tobias Kurth^1^, Imke Mayer^1^, Ana S. Oliveira Gonçalves^1^, Ricarda S. Schulz^1^, Kerstin Wainwright^1^

Aiham Alkhayer^2^, Anselm Angermaier^2^, Agnes Flöel^2^, Verena Horn^2^, Wieland Köhn^2^, Malgorzata Kotarz-Boettcher^2^, Anne Krüger^2^, Felix von Podewils^2^, Cordula Weil^2^, Carl Witt^2^,

Jean-Francois Chenot³, Simone Kiel³, Elisa Michalowsky³

Michael Böttcher^4^

Diana Graja^5^, Katrin C. Reber^5^, Olga Resch^5^

Juliane Rothe^6^, Jacqueline Syring^6^

**Affiliations**

^1^ Institute of Public Health, Charité – Universitätsmedizin Berlin, Berlin, Germany

^2^ Department of Neurology, University Medicine Greifswald, Greifswald, Germany

³ Department of General Practice, Institute for Community Medicine, University Medicine Greifswald, Greifswald, Germany

^4^ MEYTEC, Werneuchen, Germany

^5^ AOK Nordost, Potsdam, Germany

^6^ Techniker Krankenkasse, Hamburg, Germany
